# Indoor microbiota in severely moisture damaged homes and the impact of interventions

**DOI:** 10.1186/s40168-017-0356-5

**Published:** 2017-10-13

**Authors:** Balamuralikrishna Jayaprakash, Rachel I. Adams, Pirkka Kirjavainen, Anne Karvonen, Asko Vepsäläinen, Maria Valkonen, Kati Järvi, Michael Sulyok, Juha Pekkanen, Anne Hyvärinen, Martin Täubel

**Affiliations:** 1 Environmental Health Unit, National Institute for Health and Welfare, Kuopio, Finland; 2 Plant and Microbial Biology, University of California, Berkeley, California USA; 3 California Department of Public Health, Richmond, California USA; 4 School of Engineering, Aalto University, Espoo, Finland; 5 Department for Agrobiotechnology (IFA-Tulln), University of Natural Resources and Life Sciences, (BOKU), Vienna, Tulln Austria; 6 Department of Public Health, Helsinki University, Helsinki, Finland

**Keywords:** Indoor, Built environment, Moisture damage, Dampness, Indoor mold, Intervention, House dust microbiota, Amplicon sequencing

## Abstract

**Background:**

The limited understanding of microbial characteristics in moisture-damaged buildings impedes efforts to clarify which adverse health effects in the occupants are associated with the damage and to develop effective building intervention strategies. The objectives of this current study were (i) to characterize fungal and bacterial microbiota in house dust of severely moisture-damaged residences, (ii) to identify microbial taxa associated with moisture damage renovations, and (iii) to test whether the associations between the identified taxa and moisture damage are replicable in another cohort of homes. We applied bacterial 16S rRNA gene and fungal ITS amplicon sequencing complemented with quantitative PCR and chemical-analytical approaches to samples of house dust, and also performed traditional cultivation of bacteria and fungi from building material samples.

**Results:**

Active microbial growth on building materials had significant though small influence on the house dust bacterial and fungal communities. Moisture damage interventions—including actual renovation of damaged homes and cases where families moved to another home—had only a subtle effect on bacterial community structure, seen as shifts in abundance weighted bacterial profiles after intervention. While bacterial and fungal species richness were reduced in homes that were renovated, they were not reduced for families that moved houses. Using different discriminant analysis tools, we were able identify taxa that were significantly reduced in relative abundance during renovation of moisture damage. For bacteria, the majority of candidates belonged to different families within the *Actinomycetales* order. Results for fungi were overall less consistent. A replication study in approximately 400 homes highlighted some of the identified taxa, confirming associations with observations of moisture damage and mold.

**Conclusions:**

The present study is one of the first studies to analyze changes in microbiota due to moisture damage interventions using high-throughput sequencing. Our results suggest that effects of moisture damage and moisture damage interventions may appear as changes in the abundance of individual, less common, and especially bacterial taxa, rather than in overall community structure.

**Electronic supplementary material:**

The online version of this article (10.1186/s40168-017-0356-5) contains supplementary material, which is available to authorized users.

## Background

Living and working in buildings that are affected by moisture damage and dampness has been associated with adverse health outcomes, specifically respiratory symptoms and infections, and exacerbation and new onset of asthma. The epidemiological evidence supporting these associations is sound and has been reviewed and evaluated repeatedly [1–4]. A causal connection between moisture damage and the exacerbation of asthma has been recently proposed [5]. The actual causative agents and the mechanisms underlying these associations are unknown.

A multitude of exposing agents may be increased by moisture in buildings. These include biological and chemical factors, such as fungal and bacterial spores and fragments, their metabolites and mycotoxins, as well as volatile organic compounds emitted from damp building materials and structures or being produced during microbial growth [3]. Of these, exposure to microbes and their structural components and metabolites are thought to be a key contributor to adverse health effects observed in occupants of damp buildings. Moisture damage and dampness in buildings is linked to microbial growth on building materials [6–9]. Qualitative and quantitative changes in fungal and bacterial exposures in indoor air and dust in response to moisture problems have been reported repeatedly [10–17], but other studies did not find clear associations [18–21]. Studies investigating the role of microbes in the association between moisture damage and adverse health are less conclusive and have produced little consistent and often contradictory results. Both positive and negative associations, as well as the absence of an association of microbial exposure in moisture damage buildings with respiratory symptoms and clinical measurements have been reported [4, 22–26]. It is apparent that not only quantitative but also qualitative aspects of the exposure in damp buildings may be relevant, defining the needs for exposure assessment methods towards increasing the resolution to better define the qualitative content of the microbial exposure.

Our current understanding of moisture-related changes in the indoor microbiota largely relies on studies using traditional cultivation-based fungal and bacterial measurements. This work has culminated in proposing fungal and bacterial taxa which—when present in indoor air or on building materials—may indicate moisture damage and dampness problems in a building [6]. Quantitative PCR (qPCR) was introduced to the field almost two decades ago [27] and is widely used today [28, 29]. However, a limitation in qPCR-based approaches is that the target taxa are largely based on knowledge from cultivation studies.

High throughput sequencing approaches have been applied to indoor environments to improve our understanding of the microbial ecology in buildings and to ultimately assess health implications of exposure to the indoor microbiome. Only few of these sequencing studies have addressed at least to some extent the effect of moisture damage and indoor dampness on indoor microbiota [12, 30–35], and to date there are no clear patterns of microbial response to water damage.

There is need for dedicated studies that would generate new information on microbial signatures of moisture damage, utilizing microbial exposure data with high resolution. In our current study we aimed to (i) characterize fungal and bacterial microbiota in the house dust of severely moisture-damaged residential homes, (ii) identify microbial signatures of moisture damage by following homes through moisture damage renovations, and (iii) test whether the associations of the identified taxa with moisture damage are replicable in a large cohort of residential homes with varying degrees of observed moisture damage and visible mold.

## Methods

### Study population

The HOTES study (HOmeloukku ja TErveysSeuranta—“moldy homes and health study”) was conducted in severely moisture-damaged homes across Finland and among their occupants. HOTES is an intervention study with the main focus on the effects of moisture damage and moisture damage interventions on occupant exposure and health. In this current report, we focus on the microbial changes during moisture damage interventions; the health outcomes in this study will be analyzed and reported separately. Families contacted the Organization for Respiratory Health (Hengitysliitto, Finland) and requested help in assessing their homes because of possible moisture damage. Once a civil engineer visited the homes and verified severe moisture damage requiring an intervention, the occupants and their homes were recruited to the study. All participating homes were single-family houses and were located mainly in the central and eastern parts of Finland.

From 2008 to 2013, 41 severely damaged houses and their occupants were recruited to this study and pre-intervention exposure samples were collected. Of these 41 homes, a total of 20 homes were also examined post-interventions, with exposure and health assessments conducted the same way as pre-intervention, and paired pre and post house dust samples were used for the current analyses (Table 1). Homes that underwent an intervention compared to homes that did not, had more occupants and higher number of toxins detectable from house dust; no significant differences were observed for the other key environmental and microbial variables (levels of Gram-positive and Gram-negative bacteria, total fungi and FERMI index, and viable bacteria and fungi detected from building material samples; prevalence of pet keeping, living in a farming environment, urban versus rural location of the home, and season of dust sampling). Of those 20 homes, nine underwent a moisture damage renovation where the families remained in the same home following building repair (referred to here as “renovated”), eight families moved into an existing home, and three families built a new home. In our analyses, homes where families moved into an existing or a newly built home were combined and referred to as “moved.”

**Table 1:** Study populations used in the analyses, numbers of homes, and number of samples

	Moisture-damaged homes	Moisture damage intervention populations	
		Moisture damage renovations	Moved to a newly built or existing house
Number of homes	41	9	11
Number of samples			
Floor dust living room (FDLR)	40	8	11
Floor dust other room (FDOR)	24	4	1
Airborne settled dust (SD)	37	8	9
Total house dust samples	101	20	21

### Building inspections and determination of viable fungi and bacteria from building material samples

Information on building structures, history, materials and moisture damage was received from the technical investigation and interview that was performed by civil engineers with the Organization for Respiratory Health. The civil engineers took building material samples from damaged locations before the renovation to confirm microbial growth in the building structures, where that was deemed necessary. Through cultivation, viable counts and composition of microbes were determined from building material samples, as described earlier [7]. Two fungal media, 2% malt extract agar (MEA) and dichloran-glycerol 18- agar (DG18) with chloramphenicol, and one bacterial medium, tryptone yeast glucose agar (TYG) with natamycin, were used. These are commonly used and broad media for the detection of fungi and bacteria in indoor environments. MEA favors growth of hydrophilic fungi, while DG18 is more suitable for growth of xerophilic fungi [6]. The total concentration of viable fungi was based on the higher value observed in the two fungal media (MEA or DG18). The categorization of homes based on fungal and bacterial colony counts from building materials has been described elsewhere (Järvi et al., manuscript submitted). In brief, median values of viable counts of fungi and bacteria were calculated for each of the homes from all of the building material samples taken from living areas of the homes, including living rooms, bedrooms, and kitchens based on the assumption that most exposure happens in these rooms.

### House dust sampling for microbial determinations

House dust samples, representing integrated exposure over time, were collected with protocols developed and applied in previous studies [36]. Floor dust sample was obtained by attaching a nylon sample sock to a vacuum cleaner’s pipe and by vacuuming a floor area (preferably rugs) of 5 m^2^ for 10 min in the living room (FDLR). For homes where major moisture-damaged areas were observed outside the living room, vacuumed floor dust samples were collected from these other areas (FDOR). A settled dust sample (SD) was vacuumed with the same method from elevated areas above floor level, e.g., from the top of cupboards or shelves (1–2 m^2^ sampling area, typically; 10 min vacuuming). The floor dust samples were homogenized by sieving through a sterile strainer, and all samples were dried in a desiccator, aliquoted, and stored at − 20 °C until DNA extraction.

### DNA extraction

DNA was extracted from approximately 20 mg of house dust that was accurately weighted into 2-mL tubes with glass beads, starting with a bead-milling step for mechanical cell disruption [37], using MiniBeadbeater-16 for 1 min (Biospec Products Inc., USA). DNA was extracted and cleaned from the samples using the Chemagic DNA Plant–kit (PerkinElmer chemagen Technologie GmbG, Germany) and KingFisher mL DNA extraction robot (Thermo Scientific, Finland). 0.64 μg of deoxyribonucleic acid sodium salt from salmon testes (Sigma Aldrich Co., USA) [38] was added to the samples prior to extraction as an internal standard, in order to assess and correct for the presence of inhibitors and the performance of the DNA extraction. DNA was stored at − 20 °C until subsequent analysis. Negative (reagents) and positive (bacterial and fungal mock community) controls were included in the DNA extraction step along with house dust samples.

### Quantitative PCR analysis

Quantitative PCR (qPCR) was used for quantitation of fungal and bacterial biomass using previously published qPCR assays: Gram-positive and Gram-negative bacteria groups [37], and total fungal DNA [28]. QPCR analyses of the fungal species included those used for calculation of the Finnish Environmental Relative Moldiness Index (FERMI) [39] and were performed as previously described [28]. The FERMI index is a quantitative-based assessment of different types of fungi indoors modeled after ERMI [29]. QPCR analysis of the internal standard salmon testis DNA followed the instructions by Haugland et al. [38]. In the bacterial duplex assay (Gram-positive and negative bacteria), 20 μl reaction mix were used, consisting of 10 μl of Environmental Master Mix (Applied Biosystems Inc., Foster City, CA), 1.5 μl bovine serum albumin (2 mg/ml), 500 nM forward and reverse primers and 200 nM each of the two TaqMan probes, 3.7 μl of nuclease-free water (HyClone Laboratories Inc., Utah, USA), and finally 2 μl of template DNA. Reactions were performed in 0.2-ml 96-well plates (Agilent Technologies Inc., USA) for Stratagene Mx3005P QPCR System (Agilent Technologies Inc., USA) equipment. Numbers of microbial cell equivalents in the samples were calculated using relative quantification [40] and presented as detected microbial cell equivalent per milligram dust.

### Determination of microbial toxins

A 50-mg subsample of dust was used for the secondary metabolite analysis. Metabolites were extracted and diluted from dust using acetonitrile/water/acetic acid solution. The analysis was performed as described earlier [41] but with an expanded range of detectable microbial secondary metabolites (348 fungal and 44 bacterial metabolites) and a more sensitive LC-MS/MS system. In brief, we used Agilent 1290 Series HPLC System (Agilent, Waldbronn, Germany) coupled to a QTrap 5500 equipped with Turbo Ion Spray ESI source (Applied Biosystems, Foster City, CA, USA) in connection with a Gemini® C18 column, 150 × 4.6 mm i.d., 5 μm particle size protected by a C18 security guard cartridge, 4 × 3 mm i.d. (all from Phenomenex, Torrance, CA, USA). A methanol/water gradient containing 1% acetic acid and 5 mM NH4Ac was used at 1 ml/min. Data acquisition was performed in the scheduled multiple reaction monitoring (sMRM) mode in both positive and negative polarity using two separate chromographic runs per sample. Confirmation of the identity of the investigated analytes was obtained through acquiring two sMRM transitions (except for moniliformin and 3-nitropropionic acid, which yield only one detectable fragment ion) and comparison of the intensity ratio and LC retention time to an authentic standard. Quantification was performed based on linear, 1/× weighed calibration curves deriving from serial dilutions of a multi-analyte standard.

### DNA sequencing

The DNA extracted from house dust and control samples was shipped frozen to the sequencing service partner LGC Genomics (Germany), who did the library preparation and sequencing. For bacteria, a pre-amplification of sample DNA was performed using primers 341F (CCTACGGGNGGCWGCAG) [42] and 1061R (CRRCACGAGCTGACGAC) [43]. The PCRs included approximately 5 ng of DNA extract, 15 pmol of each primer in 20 μl volume of MyTaq buffer containing 1.5 units MyTaq DNA polymerase (Bioline GmbH, Luckenwalde, Germany), and 2 μl of BioStabII PCR Enhancer (Sigma-Aldrich Co.). Pre-amplification PCRs were carried out for 20 cycles using the following parameters: 2 min 96 °C predenaturation; 96 °C denaturation for 15 s, 50 °C annealing for 30 s, 70 °C extension for 90 s, hold at 8 °C. The V4 region of the 16S rRNA gene was amplified using 515F/806R primers for 20 cycles [44]. For fungi, the ITS1 region of the Internal Transcribed Spacer (ITS) was amplified using ITS1F/ITS2 primers and 33 cycles [45].

The PCRs included either approximately 5 ng of DNA extract (for fungi), or 1 μl pre-amplification product (for bacteria), 15 pmol of each forward primer 515F N_1–10_GTGCCAGCMGCCGCGGTAA and reverse primer 806R N_1–10_GGACTACHVGGGTWTCTAAT, ITS1F N_1–10_CTTGGTCATTTAGAGGAAGTAA and ITS2 N_1–10_GCTGCGTTCTTCATCGATGC (N_1–10_ indicate the 10 nucleotide inline-barcodes), in 20 μl volume of MyTaq buffer containing 1.5 units MyTaq DNA polymerase (Bioline GmbH, Luckenwalde, Germany) and 2 μl of BioStabII PCR Enhancer (Sigma-Aldrich Co.). For each sample, the forward and reverse primers had the same 10-nt barcode sequence. PCRs were carried out for either 33 cycles (ITS-PCR on DNA extract) or 20 cycles (16 s–PCR on pre-amplification product) using the following parameters: 2 min 96 °C predenaturation; 96 °C denaturation for 15 s, 50 °C annealing for 30 s, 70 °C extension for 90 s, hold at 8 °C. About 20 ng amplicon DNA of each sample were pooled for up to 48 samples carrying different barcodes. PCRs showing low yields were further amplified for 5 cycles. The amplicon pools were purified with one volume Agencourt AMPure XP beads (Beckman Coulter, Inc., IN, USA) to remove primer dimer and other small mispriming products, followed by an additional purification on MinElute® columns (QIAGEN GmbH, Hilden, Germany). About 100 ng of each purified amplicon pool DNA was used to construct Illumina libraries using the Ovation® Rapid DR Multiplex System 1–96 (NuGEN Technologies, Inc., CA, USA). Illumina libraries (Illumina, Inc., CA, USA) were pooled and size selected by preparative gel-electrophoresis.

Sequencing was performed on an Illumina MiSeq with V3 chemistry resulting in paired-end reads with a length of 300 bp each. The libraries were demultiplexed using Illumina’s bcl2fastq v1.8.4 (https://support.illumina.com/downloads/bcl2fastq_conversion_software_184.html) and all sequence reads processed with custom Python v2.7.6 scripts to sort them by sample, removing barcode and amplicon primer sequences. Adapter sequences were removed from the 3′ end of reads with a proprietary script discarding reads shorter than 100 bp.

### Bioinformatic analysis

All 16S rRNA gene and ITS-targeted amplicon reads were processed and analyzed using QIIME (Quantitative Insights Into Microbial Ecology) software version 1.9.1 [46]. The raw bacterial reads were preprocessed by removal of artificial sequences such as adapters by cutadapt software [47], followed by trimming of bad quality reads and ambiguous sequences by the Trimmomatic software [48]. Then, the preprocessed reads were merged using FLASH (Fast Length Adjustment of SHort reads) software [49]. UCHIME [50] was employed to remove chimeras in the preprocessed reads using the USEARCH algorithm [51]. After chimera removal, the preprocessed reads were aligned using pynast [52] with the Greengenes database [53] and sorted with > 97% similarity into operational taxonomic units (OTUs) using open reference OTU picking approach for bacteria. For fungi, the data processing was similar until the chimera removal step. We used FHiTINGS (Fungal High throughput Taxonomy Identification in NGS) to calculate taxa-based OTU groups instead of clustering [54]. Negative and positive (bacterial and fungal mock) controls were included in the sequence processing of the samples in order to inform estimates of alpha richness in the samples and to exclude samples closely clustering to control samples in PCoA plots. Alpha rarefaction was done at 2700 sequences for bacteria and at 737 sequences for fungi. Taxonomic classification was obtained using the RDP classifier [55] for bacteria and FHiTINGS for fungi. Alpha-diversity values were calculated in QIIME using Chao1, Simpson, and Shannon; beta-diversity values were calculated using Unifrac distance metric for bacteria [56] and the Bray-Curtis and Binary Jaccard metrics for fungal analyses. Emperor was used to visualize the beta diversity plots. The NMDS plot was done with the ggplot2 package [57] from R Version 3.0.2 [58] to display the beta-diversity differences between the samples.

A phylogenetic tree was constructed with the 3719 bacterial OTUs identified. The sequences for the OTUs were obtained from the Greengenes database and the phylogenetic tree was constructed using the Neighbor joining algorithm within the MEGA7 software [59].

### Replication study in the LUKAS cohort

Details on the LUKAS cohort, the dust sampling and sample processing, sequencing and sequence processing are provided in the Supplement (Additional file 1). In brief, the LUKAS study is a birth cohort that consists of 442 single family homes in eastern and middle Finland with approximately one fourth of the homes being farming homes [60]. Floor dust sampling and standardized home inspections were performed in early life of the participating study children, providing classification on moisture damage (no, minor, major) and visible mold (no, mold spots, visible mold) for the current analyses. Dust processing, DNA extraction, PCR and bacterial 16S rRNA gene and fungal ITS1 amplicon sequencing, sequence processing, and bioinformatics were similar between the HOTES and LUKAS datasets. More detail on the cohort, dust sampling, and sequencing is provided in Additional file 1, Supplement text.

### Statistical analyses

#### Impact of environmental and microbial factors on community composition

We used the ANOSIM statistical test available in QIIME to study associations of environmental factors with other microbial measurements, as well as the impact of moisture damage interventions on microbial community composition. Bacterial weighted and unweighted Unifrac distance and fungal Bray-Curtis and Binary Jaccard distance metrics were used to define bacterial and fungal beta-diversity. qPCR biomass determinations (Gram-positive and Gram-negative bacteria, total fungi), FERMI index, and number of detected microbial secondary metabolites were categorized into tertiles for the analyses on associations with bacterial and fungal beta-diversity. For the analyses against viable bacterial and fungal growth on building materials, pre-intervention study homes were divided into three approximate equal sized groups—low, moderate, high bacterial/fungal viable growth—based on median values derived from total viable bacterial and fungal counts in cultivation of building material samples in these homes. Pet keeping was defined as any number of dogs and/or cats. Other home environmental determinants tested were the season of sampling and urban versus rural location of the study home (the metadata file used in these analyses is supplemented in Additional file 2, Table S1).

#### Ιmpact of interventions on alpha-diversity and top abundant taxa

The impact of moisture damage interventions—for all interventions combined and for “renovation” and “families moved” separately—on Chao1 estimated richness, Shannon diversity index, and the 15 most abundant bacterial and fungal genera (selected based on their median prevalence in samples pre intervention) were done in pairwise comparisons of samples taken in the same locations pre and post intervention (information on the paired sample codes is provided in Additional file 2, Table S2). Non-parametric statistical methods were used because outcomes were not normally distributed. The differences between pre and post were tested using Wilcoxon signed-rank test (for matched pair data) using SAS software (version 9.3, SAS Institute Inc., Cary, NC, USA).

#### Identification of indicator taxa

We used three different approaches in an attempt to identify bacterial and fungal indicator taxa associated with moisture damage renovations. Approach 1 is a community structure-based approach, identifying principal coordinates from the community distance metrics with associations to the outcome of interest and selecting those taxa correlating strongest with these coordinates; approach 2 is a commonly used tool for biomarker discovery, however, not allowing for adjustment of potential confounders, which approach 3 adds to the analyses.

In approach 1, we first used the principal coordinate (PC) scores derived from bacterial weighted and unweighted Unifrac distance and fungal Bray-Curtis and Binary Jaccard distance metrics. We considered PCs with Eigenvalue > 1 for the analysis. Associations of the selected PCs with moisture damage interventions—performed separately for “renovation” and “families moved”—were analyzed using pairwise Wilcoxon signed-rank test as described above for alpha-diversity. PCs with significant associations (*p* < 0.05) were selected and Spearman rank-order correlations estimated with the relative abundance of all bacterial OTUs and fungal taxa in the sample. Correlation scores > |0.5| for bacterial and > |0.4| for fungal taxa were used as criteria to select the taxa for subsequent indicator analyses.

In approach 2, we applied LEfSe version 1.0.7 [61], which is a biomarker discovery tool based on linear discriminant analysis. LEfSe is a three-step algorithm that lists differential features with statistical and biological significance (LDA effect size). Here, we applied LEfSe to compare relative abundance of bacterial OTUs and fungal taxa in pre versus post renovation comparisons of house dust samples and to identify discriminative features. We used standard criteria of significance < 0.05 and LDA effect size > 2 to include identified taxa for subsequent analyses.

Finally, in approach 3, we applied edgeR (glm-edgeR) [62]. edgeR is a tool that has been developed for differential gene expression analysis and gene marker identification based on generalized linear model and probability distribution model fitting. The function glm-edgeR() has inbuilt false discovery rate (FDR) correction to provide corrected *p* values in the final results. Here, we use EdgeR on bacterial and fungal taxa instead of genes with the aim to identify microbial markers associated with moisture damage renovation. The analyses were adjusted for the potential confounders: sample type, season, pet keeping, urban and rural location of the home, and the number of occupants. We used a cut-off of FDR corrected *p* values < 0.1 for inclusion of taxa for further studies.

Results from approaches 1–3 were considered together. Bacterial OTUs that were identified by at least two methods and fungal taxa that were identified by any one method were considered potential indicator taxa and tested pre versus post comparisons for “renovation” and “families moved” in pairwise Wilcoxon signed-rank test.

#### Replication study in the LUKAS cohort

Kruskal-Wallis test with Bonferroni correction for testing multiple candidate OTUs was used to study whether the OTUs associated with moisture damage in HOTES were found in higher levels in LUKAS homes with than without moisture damage or visible mold. The independence of significant differences of potential confounders (LUKAS cohort, type of living area (rural farm, rural non-farm, suburban), construction year of the house, building type (single family house, others), heating (central water-heating, electric heating), and season of dust collection (fall, winter, spring, summer) were tested with quantile regression using 200 permutations to obtain 95% confidence intervals.

## Results

### Microbiota of different types of house dust in moisture-damaged homes

Sample type, i.e., floor dust (FDLR) versus settled dust (SD), had a small but significant impact on both bacterial (*R* = 0.054; *p* = 0.01) and fungal (*R* = 0.096; *p* = 0.001) abundance weighted communities in ANOSIM analyses, and we therefore explored the effects of moisture damage separately for living room floor and airborne settled dust (detailed below). The mean relative abundances of the most prominent bacterial and fungal genera in the different sample types are presented in Additional file 1 (supplemental text) and Additional file 3: Figures S1A and S1B. Bacterial and fungal alpha-diversity estimates (Chao1 estimates richness, Shannon index) were not significantly different between the different sample types (Additional file 2: Table S3).

We evaluated associations of environmental variables and other microbial measurements on the beta-diversity of bacterial and fungal microbiota in house dust of moisture-damaged homes. Table 2 presents results based on ANOSIM analyses using weighted bacterial Unifrac and fungal Bray-Curtis distance-based calculations. The results for using both weighted and unweighted bacterial and fungal distance matrices are detailed in Additional file 2: Tables S4 and S5). Overall, effect sizes (*R* value) were small. Household environmental characteristics more strongly influenced fungi than bacteria in homes. Urban versus rural location of the home showed a statistically significant but small overall impact for fungal microbiota in the settled dust (Table 2). The season of sampling also had an impact on fungal communities in the floor dust (Table 2), as well as on the bacterial unweighted Unifrac distance (Additional file 2: Table S4). Interestingly, pet keeping appeared not to affect house dust microbiota in this study population.

**Table 2:** Impact of home environmental and microbial measurement parameters on the composition of the bacterial and fungal microbiota in severely moisture-damaged homes. ANOSIM analysis used weighted Unifrac distance for bacteria and fungal Bray-Curtis distance (FDLR, floor dust living room; SD, settled dust)

	Bacterial weighted Unifrac						Fungal Bray-Curtis					
	FDLR			SD			FDLR			SD		
Variable tested	*N*	*R*	*p* value	*N*	*R*	*p* value	*N*	*R*	*p* value	*N*	*R*	*p* value
Environmental determinants												
Pets	38	0.020	0.221	35	−0.003	0.466	38	−0.050	0.985	34	0.043	0.115
Season of sampling	39	0.014	0.348	34	−0.012	0.587	39	0.205	*0.001*	33	0.049	0.142
Urban vs. rural	40	−0.119	0.971	36	0.105	0.108	40	0.128	0.067	35	0.162	*0.018*
Biomass—qPCRs												
Total fungi	39	0.052	0.205	34	0.017	0.330	39	−0.012	0.529	33	−0.014	0.550
Gram-negative bacteria	39	−0.008	0.567	34	−0.009	0.518	39	−0.046	0.828	33	0.038	0.266
Gram-positive bacteria	39	−0.047	0.807	34	0.050	0.189	39	−0.047	0.792	33	−0.020	0.560
FERMI qPCR index	39	0.117	*0.035*	34	<0.001	0.444	39	0.098	0.054	33	0.026	0.315
Microbial secondary metabolites												
Number of microbial “toxins”	39	0.054	0.163	28	−0.056	0.776	39	0.034	0.273	28	−0.050	0.733
Viable microbes in BM												
Bacterial growth in BM	36	−0.053	0.702	31	0.352	*0.004*	36	0.000	0.467	30	0.007	0.483
Fungal growth in BM	36	−0.023	0.665	31	−0.042	0.827	36	0.090	*0.035*	30	0.150	*0.010*

Significant *p*-values <0.05 are shown in italics

We also compared other microbial measurements with the sequence-based community approach, and as a whole found modest links between them. Categorized groups of total fungal and bacterial biomass (determined with qPCR) and number of different microbial secondary metabolites (determined with LC-MS/MS) did not have significant impact on either bacterial or fungal communities (Table 2). However, the Finnish Relative Moldiness Index [39] correlated significantly with the bacterial and borderline significantly with the fungal microbiota in floor dust. Notably, the severity of bacterial and fungal growth in the study homes—determined as viable microbial counts from building material samples—was linked to the bacterial and fungal microbiota in house dust (Table 2). That is, those homes that had different levels of microbial growth on building materials (high/medium/low) tended to have different microbial community composition, although the data do show noise around this trend (Additional file 4: Figure S2).

### The impact of moisture damage interventions on house dust microbiota

We followed 20 homes/families through moisture damage interventions, which in nine homes involved remediation of the moisture damages in the course of renovations, and in the case of 11 homes, families moved (Table 1). We monitored the impact of the interventions in pre versus post comparisons in these 20 homes, in terms of the numbers and types of bacterial and fungal taxa, in order to investigate how interventions changed potential microbial exposures.

**Fig. 1:**
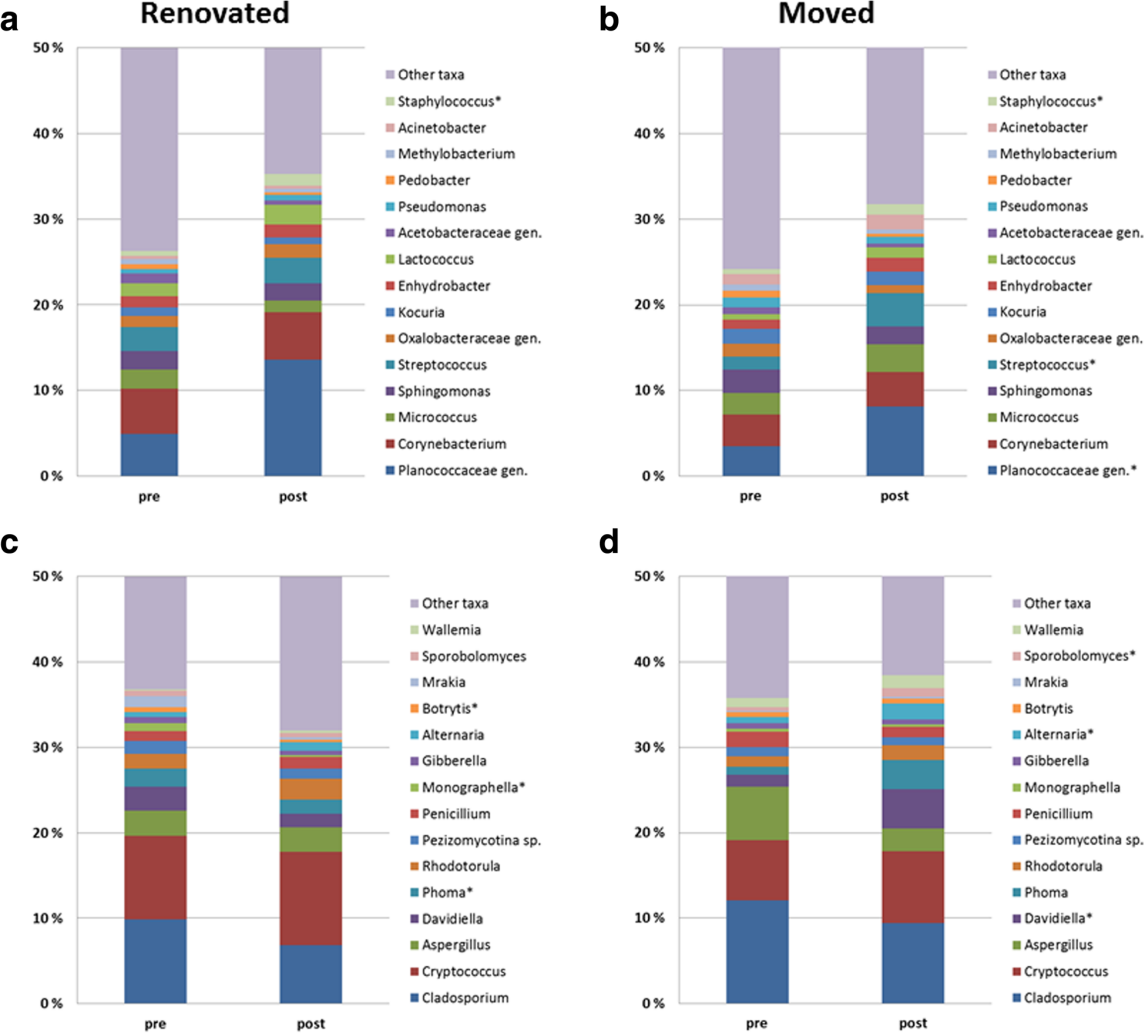
Median relative abundance of the 15 top abundant bacterial (**a**, **b**) and fungal (**c**, **d**) genera in house dust of moisture-damaged homes: pre and post moisture damage renovations (20 + 20 paired house dust samples; bacteria 1A, fungi 1C), and pre and post moisture damage interventions by moving into another house (21 + 21 paired house dust samples; bacteria 1B and fungi 1D). *Significant (*p* < 0.05) differences of relative abundance of taxa in pairwise sample comparison pre vs. post using Wilcoxon signed-rank test

In pairwise sample comparisons (pre versus post interventions) of the relative abundance of the 15 top most abundant bacterial and fungal genera in the house dust samples, we found little significant changes (Fig. 1). For bacteria, we observe significant increases in the relative abundance of *Staphylococcus* after moisture damage renovations, and of *Staphylococcus*, *Streptococcus*, and *Planococcaceae* genera in house dust after families had moved to a new home. For fungi, we found significant decreases for *Phoma*, *Bortrytis*, and *Monographella* genera during moisture damage renovations and significant increase of *Davidiella*, *Sporobolomyces*, and *Alternaria* when people moved to another home.

**Fig. 2:**
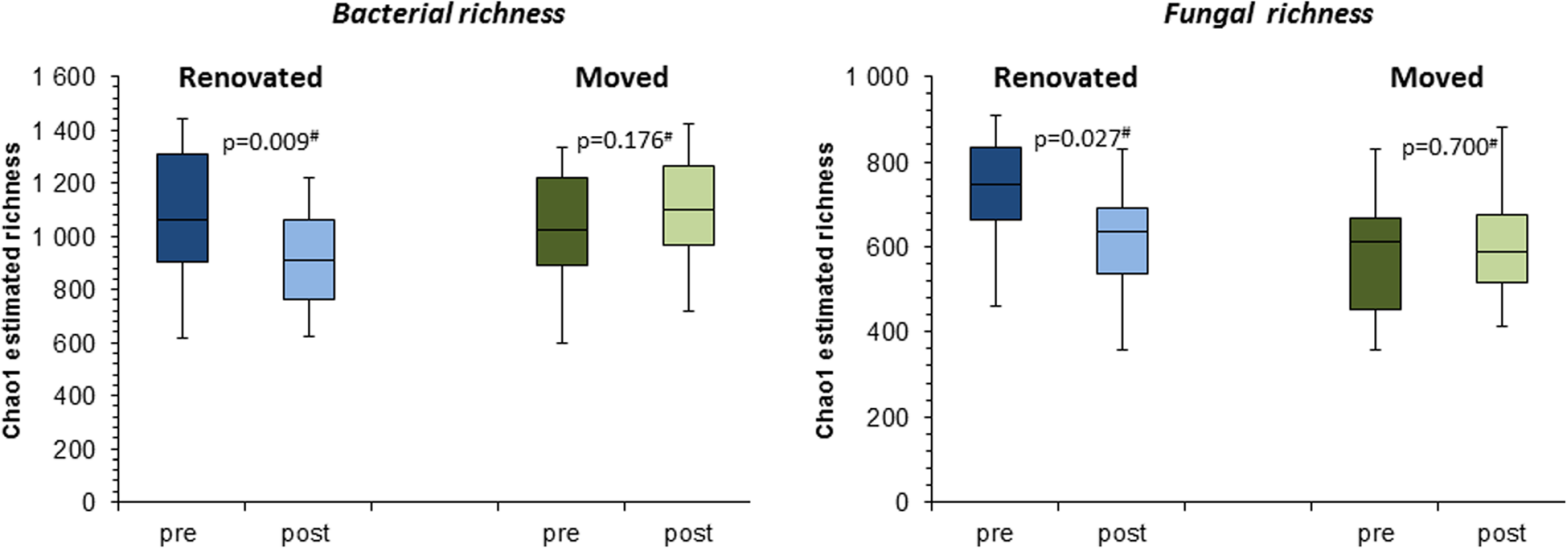
Impact of moisture damage interventions—i.e., *renovation* of moisture damage or families *moving* to another existing or newly built home—on bacterial and fungal Chao1 richness (data presented refer to 9 homes (20 house dust sample pairs) pre versus post renovation, and for 11 homes (21 sample pairs) pre versus post moving). ^#^ *p* value based on pairwise sample comparison in Wilcoxon signed-rank test)

We observed a significant reduction in the Chao1-estimated richness of both bacterial and fungal taxa in house dust for moisture damage renovations. We did not observe a similar reduction in microbial richness in the cases where families moved (Fig. 2). Results were similar when considering Shannon diversity index (Additional file 2: Table S6).

**Fig. 3:**
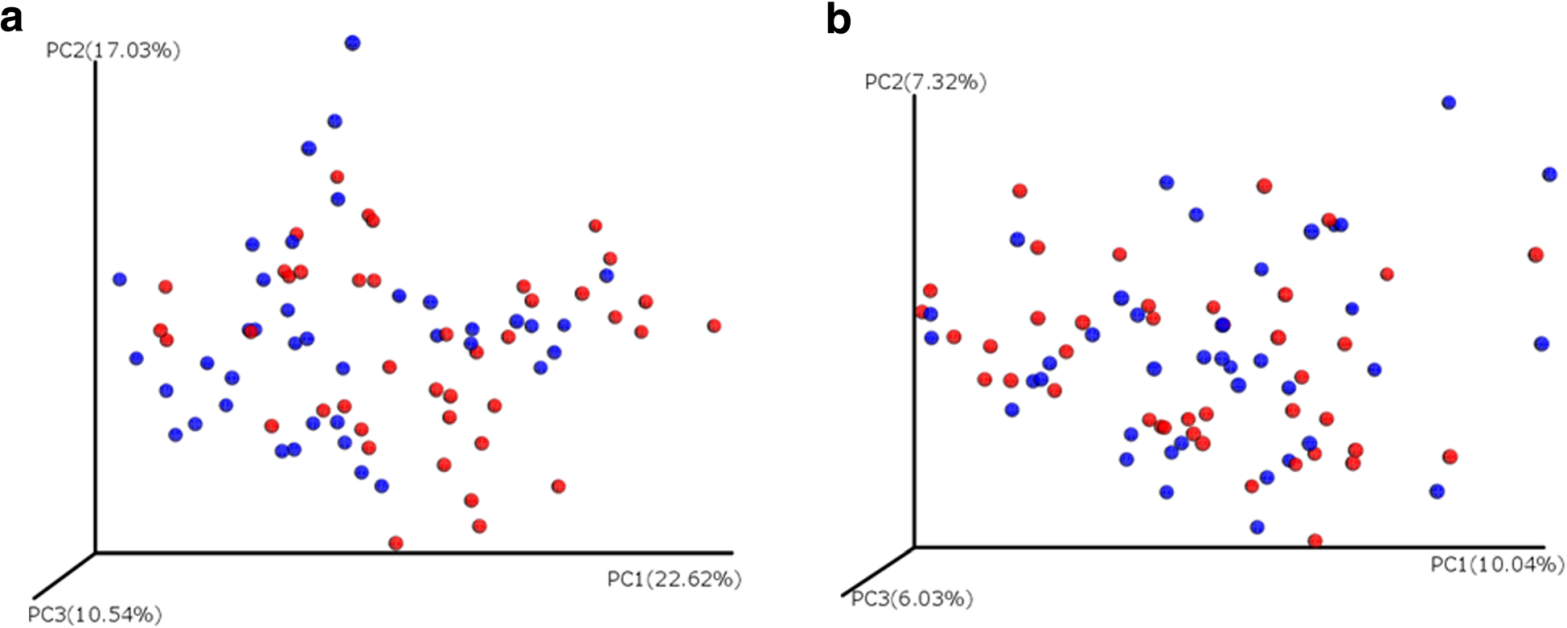
Bacterial (**a**) and fungal (**b**) community composition (abundance weighted beta-diversity, PCoA plots) in house dust samples of moisture-damaged homes pre and post interventions (pre intervention: red; post: blue)

Moisture damage intervention had only a very subtle (*R* values below 0.1) though significant impact on the bacterial community composition when measured by weighted Unifrac distance (Table 3). Similarly, renovation had a small but significant impact on the fungal community composition as measured by the Binary Jaccard distance (Table 3). Figure 3 shows the bacterial and fungal communities pre and post moisture damage interventions.

**Table 3:** Impact of moisture damage interventions on weighted and unweighted bacterial and fungal beta-diversity (analyzed with ANOSIM). Results are shown for all interventions together (39 pre 41 post samples; note: two house dust samples collected in one home served as pre samples for both, renovation and moving, as part of the family moved, while the other part renovated the existing home) and for moisture damage renovations (20 sample pairs) and cases where families moved (21 sample pairs) separately

		Bacteria				Fungi			
		Weighted Unifrac		Unweighted Unifrac		Bray-Curtis		Binary Jaccard	
Study population	*N*_samples_	*R*	*p* value	*R*	*p* value	*R*	*p* value	*R*	*p* value
All home pre and post	39 + 41	0.043	*0.018*	0.024	0.060	0.008	0.233	0.015	0.137
Renovated homes pre and post	20 + 20	0.042	0.102	0.017	0.235	−0.003	0.481	0.061	*0.029*
Homes where family moved pre and post	21 + 21	0.045	*0.043*	0.024	0.154	0.028	0.093	0.026	0.144

Significant *p*-values <0.05 are shown in italics

### Identification of indicator taxa

Bacterial and fungal taxa that were associated with moisture damage *renovations* in buildings were identified by applying various statistical approaches.

First we identified principal coordinates based on weighted bacterial Unifrac and fungal Bray-Curtis distance with Eigenvalues above 1 that associated with moisture damage renovations (Additional file 2: Table S7). PCoA1 for bacteria and PCoA7 for fungi were selected based on their associations. The 157 bacterial and 33 fungal OTUs, respectively, that correlated significantly (*p* < 0.05) and the strongest (Spearman rank-order Correlations rho > |0.5| for bacteria and > |0.4| for fungi) with those two axes were selected as potential indicator taxa (Additional file 2: Tables S8 and S9). For bacteria, almost all of these selected OTUs were reduced in relative abundance during moisture damage renovations and were largely allocated within the *Actinomycetales* order. For fungi, we also observed mostly decreases in the relative abundance of the selected taxa.

Second, we performed indicator taxa analyses using LEfSe. Fifty-two bacterial and 10 fungal taxa were identified, applying the generally recommended criteria of *p* value < 0.05 and a LDA effect size > 2 (Additional file 2: Tables S10 and S11). For bacteria, most of these OTUs were decreased in relative abundance during moisture damage renovations and were again frequently allocated to the *Actinomycetales* order. For fungi too, abundance of the identified taxa were more often reduced during moisture damage renovations; fungal taxa were allocated to various phylogenetic groups, mostly within the Ascomycota and Basidiomycota phyla.

Third, we applied glm-edgeR for the identification of indicator taxa, adjusting for sample type, season, pet keeping, the number of residents in the homes, and rural/urban location of the study homes. Applying a criterion of FDR corrected *p* values < 0.1, we identified 30 bacterial OTUs and 6 fungal indicator taxa with this approach (Additional file 2: Tables S12 and S13).

**Fig. 4:**
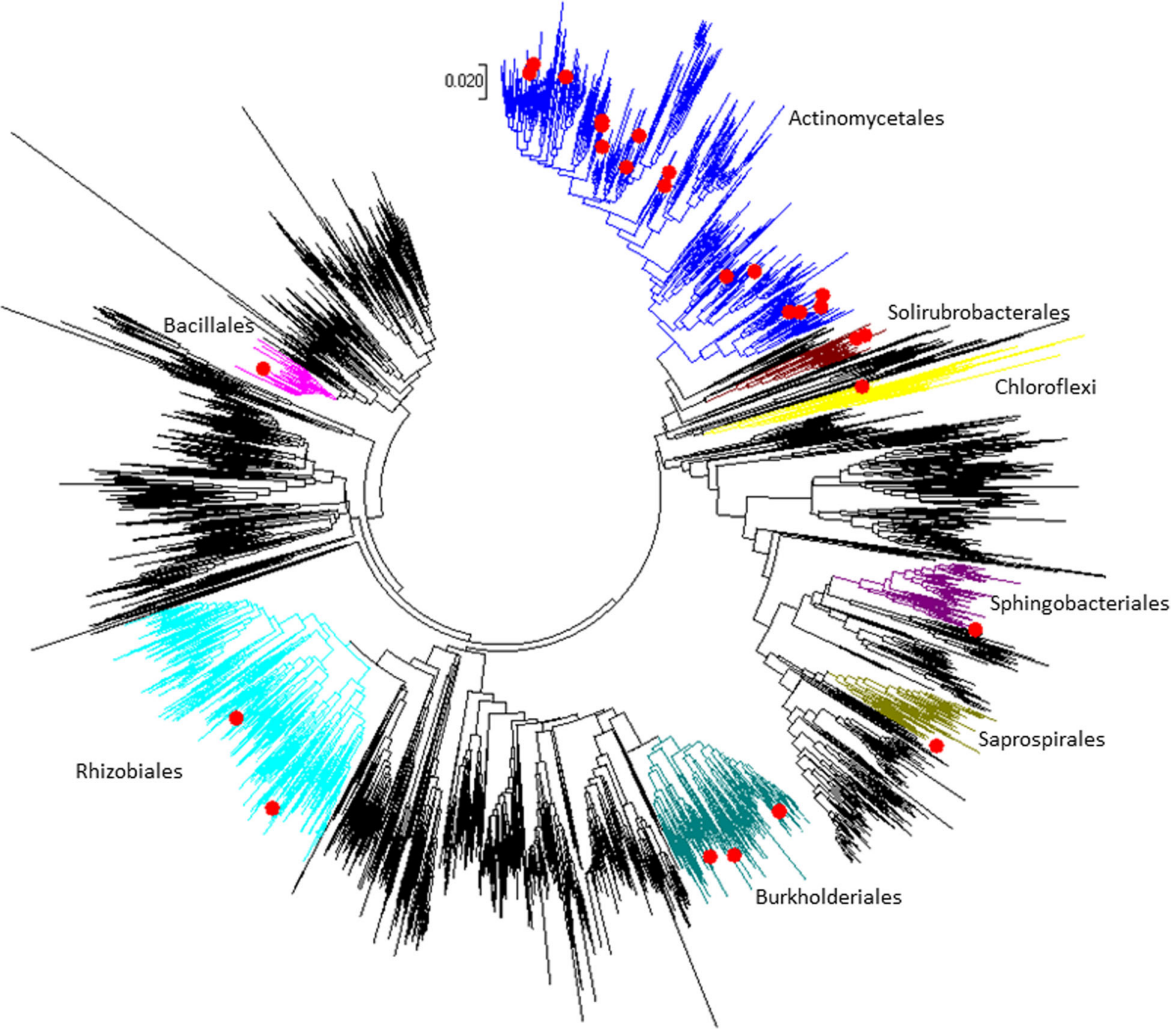
Visualization of the phylogenetic allocation of bacterial candidate OTUs that were statistically associated with moisture damage renovations in this study (marked as red dots). The phylogenetic tree was constructed with 3719 bacterial OTUs found in the current study in Finnish homes pre and post moisture damage interventions. The sequences for the OTU IDs included here were obtained from the Greengenes database and the phylogenetic tree was constructed using Neighbor joining algorithm using MEGA7 software [59]. Twenty-seven candidate OTUs and their taxonomy at the level of order (in one case phylum) are highlighted

Finally, we created overlap tables for the bacterial and fungal taxa identified via the different approaches (Additional file 2: Tables S14 and S15) and selected the most promising candidate taxa based on their repeated detection via different approaches. For bacteria, we identified 27 OTUs that were picked up with at least two of the three methods (Table 4 and Additional file 2: Table S14). For fungi, none of the taxa were identified with more than one approach, resulting in a total of 49 taxa that were highlighted as potential moisture damage indicators (Additional file 2: Table S15). In order to confirm the validity of the taxa that were identified, we subsequently tested these 27 bacterial and 49 fungal candidate taxa individually in the HOTES dataset, exploring changes in their relative abundance and their association with moisture damage interventions, including renovation and in addition moving to a new home. Of the 27 bacterial OTUs tested, 25 showed significant (*p* < 0.05) associations with moisture damage renovations, all except a *Staphylococcus* OTU being reduced in relative abundance in house dust after renovation activities (Table 4). The majority of bacterial OTUs that associated with moisture damage renovations were allocated to various families within the *Actinomycetales* order, as highlighted in the phylogenetic tree in Fig. 4. Six of these OTUs were also associated with moving as the mean of moisture damage intervention (Table 4). For fungi, 11 of the 47 tested taxa were significantly (*p* < 0.05) associated with moisture damage renovations (nine reduced in relative abundance, two increased: *Rhodotorula buffonii* and *Tremellaceae*) (Table 5). Two of those taxa, *Humicola nigrescens* and *Mortierella umbellata*, showed also borderline significant (*p* < 0.1) association with moving.

**Table 4:** Bacterial taxa associated with moisture damage renovations, as determined via PCoA approach, LefSe, and glm-edgeR (presented are OTUs that were detected with at least two of the three approaches; arrows indicate decrease or increase in relative abundance during moisture damage renovations)

OTU ID	Taxonomic allocation		Weighted UniFrac	LefSe	glm-edgeR	
^a^826144	Sphingobacteriaceae	Pedobacter	x	x	x	↓
^a^972343	Nakamurellaceae		x	x		↓
^a,b^965853	Solirubrobacterales		x	x		↓
^a,b^939252	Staphylococcaceae	Staphylococcus	x	x		↑
^a^876170	Microbacteriaceae	Salinibacterium	x	x		↓
^a^870223	Nakamurellaceae		x	x		↓
^a^825183	Chitinophagaceae		x	x		↓
^a^810959	Intrasporangiaceae	Phycicoccus	x	x		↓
^a^672144	Comamonadaceae	Roseateles	x	x		↓
^a^662915	Aurantimonadaceae		x	x		↓
^a^581286	Microbacteriaceae		x	x		↓
^a^549557	Nocardioidaceae		x	x		↓
^a,b^538111	Intrasporangiaceae		x	x		↓
^a^4398116	Cellulomonadaceae	Actinotalea	x	x		↓
^a,b^367851	Propionibacteriaceae	Microlunatus	x	x		↓
^a^324217	Nocardioidaceae	Aeromicrobium	x	x		↓
285591	Solirubrobacterales		x	x		↓
^a,b^279515	Intrasporangiaceae	Phycicoccus	x	x		↓
^a^207885	Burkholderiaceae	Burkholderia		x	x	↓
^a^196652	Burkholderiaceae	Burkholderia		x	x	↓
^a^134121	Microbacteriaceae		x	x		↓
^a^112867	Chloroflexi	Ellin6529	x	x		↓
^a^1105814	Bradyrhizobiaceae		x	x		↓
^a^1079481	Pseudonocardiaceae	Pseudonocardia	x	x		↓
^a,b^1051744	Microbacteriaceae		x	x		↓
1039041	Sporichthyaceae		x	x		↓
^a^1033426	Nocardioidaceae	Nocardioides	x	x		↓

^a^Statistically significant difference (*p* < 0.05; Wilcoxon signed-rank test) in house dust pre versus post moisture damage renovation in pairwise sample comparison (*N* = 20 + 20)

^b^Statistically significant difference (*p* < 0.05; Wilcoxon signed-rank test) in house dust samples pre versus post moving into a new home pairwise sample comparison (*N* = 21 + 21)

**Table 5:** Fungal taxa associated with moisture damage renovations, as identified via PCoA approach, LefSe, and glm-edgeR, and confirmed in pairwise sample comparison pre and post moisture damage renovations (*p* < 0.05 in Wilcoxon signed-rank test). Arrows indicate decrease or increase in relative abundance during moisture damage renovations

Taxonomic allocation					Bray-Curtis distance	LefSe	glm-edgeR	
Phylum	Class	Family	Genus	Species				
Ascomycota	Taphrinomycetes	Taphrinaceae	Taphrina	padi	x			↓
Basidiomycota	Agaricomycetes	Meruliaceae	Bjerkandera	adusta	x			↓
Ascomycota	Sordariomycetes	Chaetomiaceae	Humicola	nigrescens		x		↓
Ascomycota	Dothideomycetes	Venturiaceae	Venturia	chlorospora		x		↓
Zygomycota	Incertaesedis	Mortierellaceae	Mortierella	umbellata		x		↓
Basidiomycota	Microbotryomycetes	Incertaesedis	Rhodotorula	buffonii		x		↑
Basidiomycota	Tremellomycetes	Tremellaceae				x		↑
Ascomycota	Sordariomycetes	Amphisphaeriaceae	Monographella	nivalis		x		↓
Ascomycota	Sordariomycetes	Nectriaceae	Nectria	ramulariae		x		↓
Basidiomycota	Agaricomycetes	Coniophoraceae	Coniophora	puteana			x	↓
Basidiomycota	Agaricomycetes	Polyporaceae	Trametes	versicolor			x	↓

### Evaluation of indicator taxa against varying degrees of moisture damage and visible mold in homes of the LUKAS cohort

In the final step of our analyses, we aimed to test the suitability of using relative abundance data of the taxa identified in HOTES to predict moisture damage in buildings. For this purpose, we used living room floor dust microbiota data from the LUKAS cohort consisting of approximately 400 homes. We studied associations of the bacterial and fungal taxa that were identified in the HOTES study with three-level moisture damage and visible mold variables in the LUKAS homes.

Nine of the 25 bacterial OTUs identified in HOTES (Table 4) were not found in the LUKAS house dust dataset; eight OTUs showed tendencies (*p* value < 0.1) for an increase in relative abundance in house dust of homes with more severe moisture and/or mold damage (Additional file 2: Table S16). Of those, two OTUs—taxonomically allocated to *Phycicoccus* and *Aeromicrobium* taxa—were significantly (*p* value < 0.05) associated with moisture and mold damage severity in LUKAS homes after correction for multiple testing (Table 6).

**Table 6:** Replication study in the LUKAS cohort: bacterial OTUs and fungal taxa identified in the HOTES study, with significant differences (*p* < 0.05; Kruskal-Wallis test after Bonferroni correction) in relative abundance in house dust of homes categorized by moisture damage severity and extent of visible mold in the LUKAS cohort (75th Pctl, 75th percentile; moisture damage: 0 … no moisture damage, 1 … minor moisture damage, 2 … major moisture damage; visible mold: 0 … no visible mold, 1 … spots of visible mold, 2 … visible mold; *N*, number of homes)

				Moisture damage					Visible mold				
	Taxonomy					Relative abundance					Relative abundance		
OTU ID	Family	Genus	Species		*N*	Median	75th Pctl	*p* value		*N*	Median	75th Pctl	*p* value
Bacterial OTUs													
324217	Nocardioidaceae	Aeromicrobium		0	116	8.74 × 10^−5^	2.65–10^−4^		0	251	1.09 × 10^−4^	2.96 × 10^−4^	
				1	151	1.07 × 10^−4^	2.49 × 10^−4^		1	61	1.31 × 10^−4^	2.93 × 10^−4^	
				2	142	2.37 × 10^−4^	4.59 × 10^−4^	*<0.001*	2	97	2.07 × 10^−4^	3.93 × 10^−4^	*0.214*
279515	Intrasporangiaceae	Phycicoccus		0	116	0	1.55 × 10^−4^		0	251	0	1.67 × 10^−4^	
				1	151	7.02 × 10^−5^	2.44 × 10^−4^		1	61	7.94 × 10^−5^	2.06 × 10^−4^	
				2	142	9.11 × 10^−5^	3.37 × 10^−4^	*0.349*	2	97	1.31 × 10^−4^	4.14 × 10^−4^	*0.007*
Fungal taxa													
–	Chaetomiaceae	Humicola	nigrescens	0	111	0	3.80 × 10^−4^		0	247	0	5.28 × 10^−4^	
				1	149	5.28 × 10^−5^	6.31 × 10^−4^		1	56	5.97 × 10^−5^	4.49 × 10^−4^	
				2	136	2.45 × 10^−4^	8.82 × 10^−4^	*0.014*	2	93	3.57 × 10^−4^	1.22 × 10^−3^	*<0.001*

Significant *p*-values <0.05 are shown in italics

Three of the 11 fungal taxa identified in HOTES were not found in the LUKAS house dust dataset (Additional file 2: Table S17). Of the relative abundance of eight fungal taxa tested only one—that of *Humicola nigrescens*—was significantly associated with increasing moisture damage and mold severity (*p* values 0.014 and < 0.001, respectively; Table 6). The association between the higher relative abundance of *Aeromicrobium* and *Humicola nigrescens* with the increasing severity of moisture damage and between higher relative abundance of *Phycicoccus*, *Aeromicrobium*, and *Humicola nigrescens* and the increasing severity of visible mold growth was independent of potential confounders as indicated by quantile regression analysis (Additional file 5: Figure S3).

## Discussion

In this study, we utilized a cohort of severely moisture damaged homes to study the characteristics of house dust microbiota and observed a significant, though subtle, effect of viable microbial growth in building materials on the house dust microbiota composition in these homes. We followed a subsample of those homes through moisture damage renovations and showed individual microbial taxa, rather than overall community composition, to respond to these interventions. We see consistent responses mostly in bacterial taxa, specifically located in a cluster of families within the *Actinomycetales* order. Testing the potential usefulness of these taxa for indicating moisture problems in buildings in another, larger study, we find dose-response relationships in a number of taxa.

One of the key findings of our current study is that moisture damage interventions neither appear to be linked to major changes in the most abundant bacterial and fungal taxa in house dust, nor to major changes in the overall community structure. While there were some statistically significant shifts in community composition, the effect size was small and more pronounced for bacteria. Likewise, looking at changes in relative abundance in the top 15 most abundant taxa between pre and post interventions, constituting close to 50% of bacterial and more than 50% fungal sequences, we observe little significant changes. In line with these findings, two earlier studies that have considered moisture damage indicators reported similarly non-significant or barely significant associations of the microbial community composition in house dust with observations of visible mold, and the absence of associations with observations of water leaks [31, 32]. A very clear difference in both bacterial and fungal community composition in response to moisture damage was reported in a study that monitored previously flooded and non-flooded homes in Colorado [12]. It is clear that flooding represents a massive event, compared to a water leak or other moisture damage in a home. Given that the monitoring of those homes was conducted only 3 months after the flooding event, it is conceivable that the striking impact on the microbial composition in those buildings still persisted.

In spite of the absences of clear changes in community composition in our study, we did observe a significant reduction in both bacterial and fungal richness and diversity in house dust after renovation of moisture damage. Interestingly, we did not observe a similar effect in cases where people moved away from their old, damaged to a new or existing home. One could hypothesize that especially moving into an existing home would mean adding the microbiome associated with the family to the existing building microbiota, which could compensate for a loss in richness due to moving from a “wet” to a “dry” house. It is also possible that intensive cleaning following the renovation activities may account for the impact on reducing microbial richness in house dust reservoirs. However, an effective removal of moisture-damaged materials and the source of microbial growth could explain our observation. In other studies, higher fungal, but not bacterial richness has been reported for homes where water leaks had been observed compared to homes without such observations [31, 32].

Taken together, these findings suggest that the fungal and bacterial microbiota in house dust of moisture-damaged homes are not marked by a common and striking shift relative to dry homes but rather may experience more subtle changes in some of the less-abundant taxa.

### The need to identify microbial signatures in moisture-damaged residences

There are many reasons to try to identify and specify key microbial factors that are associated with moisture damage in buildings. For one, identifying these factors will allow more specific analyses of health effects upon exposure, ultimately aimed at improving our understanding of the mechanisms and causally involved agents underlying the well-established association between moisture damage in buildings and adverse health outcomes in building residents. So far, the evidence to confirm the general involvement of microbial exposure in adverse health effects in damp building has been inconclusive [4, 23, 24, 31]. There is great need for objective measurements: (i) to support building inspections for moisture damage and dampness, as a diagnostic tool; (ii) for monitoring the microbial status of buildings, flagging conditions or changes in conditions that may be adverse to human health; and ideally (iii) for grading “severity” of moisture damage in the existing building stock. It is obvious that fixing all moisture damage in buildings immediately is an impossible task, given the estimates of 10 to 50% of the existing building stock being affected by some level of moisture damage [2, 3]. Tools that would separate severe—as in more health hazardous—from less-severe moisture damage would allow prioritizing remediation efforts. Microbial measurements could support answering the needs listed here, in contributing to a holistic building assessment approach. Novel sequencing-based technology allows for a characterization of microbiota in samples with high resolution, not restricted to the fraction of taxa that can be cultivated under standard laboratory conditions. There is hope that novel, sequencing-based technologies could complement and strengthen microbial determinations in building assessments, which to date still mostly rely on cultivation technique. Thus, a major objective of this current study was to make a first step towards closing this gap, by trying to identify “signatures of moisture damage” in house dust microbiota.

### Particular taxa as indicators of moisture damage

We set out to address our goal by following severely moisture-damaged homes through moisture damage renovations and by screening for taxa that would be significantly reduced in relative abundance during these renovations. In examining the 15 most abundant genera (Fig. 2), the only significant change that we observed for bacteria was an increase of the human skin-associated bacterial taxon *Staphylococcus* in the post-renovation situation. For fungi, we did observe significant reductions in relative abundance of some of the more abundant genera during moisture damage renovations, namely *Phoma*, *Botrytis*, and *Monographella*. All three of these fungal genera are largely described in the context of being saprophytic or plant pathogenic taxa. *Phoma* has been noted in moisture-damaged buildings [9, 34, 35, 63], although it has also been detected in non-damaged buildings [63, 64]. *Botrytis* has only occasionally been reported in samples of indoor air [65, 66] and house dust of moisture-damaged buildings pre and post intervention [35]. This fungal genus is absent or only very rarely detected in samples of moisture-damaged building materials [7, 8], but it does have potential to proliferate on gypsum board [67]. We are not aware of reports of *Monographella* in indoor samples, except for a mentioning of the detection of *Microdochium*, its telemorph, in house dust of a moisture-damaged building [35].

In looking at the community as a whole rather than the most abundant taxa, we applied various methods to try to identify indicator taxa. For bacteria, we identified 25 OTUs that were picked-up by at least two of the three approaches applied and that were confirmed to be significantly associated with moisture damage renovations when tested individually using pairwise sample comparisons. The majority of these OTUs clustered within a number of families within the *Actinomycetales* order, such as *Nakamurellaceae*, *Intrasporangiaceae*, and *Nocardiaceae*, which include spore-forming, filamentous-growing taxa. Based on cultivation studies, the presence of actinobacteria in samples of indoor air and building materials has been proposed to be indicative of moisture problems in Finnish buildings [19]; the reference here is specifically to spore-forming, filamentous actinomycetes type actinobacteria, morphologically recognizable under the microscope. Also other studies have reported the presence of such bacterial taxa in moisture- and mold-damaged building materials [7, 20, 68–70]. Thus, both cultivation and cultivation-independent techniques indicate that representatives of *Actinomycetales* may be important components of moisture-damaged buildings.

The indicator taxa analysis for fungi did show much less clear patterns compared to the bacterial analysis. This was in spite of the fact that we were able to observe an impact of viable fungal growth in building materials on the mycobiota composition in house dust. The absence of clear fungal signals is striking because conditions of moisture damage, dampness, and indoor mold are in particular considered a *fungal* phenomenon, even if the relevance of bacteria in these conditions has been well recognized [9]. Based on this study, it appears to be more challenging with the current methods and their strengths and limitations to identify fungal than bacterial taxa in house dust that are associated with moisture conditions and microbial growth on and in building structures. Future studies should consider coupling house dust investigations with sequencing of moisture-damaged materials in the respective buildings, which could facilitate a more thorough search for indicator taxa.

In a replication study, utilizing house dust samples of over 400 Finnish homes that have been thoroughly inspected for observations of moisture damage and visible mold, two of the bacterial and one of the fungal potential indicator taxa were found to be associated significantly with severity of the dampness observations in these homes. Those were bacterial OTUs taxonomically allocated to *Phycicoccus* and *Aeromicrobium*, and the fungal species *Humicola nicgrescens*. None of these candidates have earlier been specifically named and linked to moisture conditions in buildings, but they have been isolated from different environmental samples. However, both of the bacterial candidates are actinobacteria and belong to the *Actinomycetales* order, which again links to the earlier notion that these sequencing-based findings appear to complement cultivation-based knowledge on the link of certain groups of actinomycetes with damp buildings. *Humicola* is a mostly soil-associated fungus, but has been reported occasionally in indoor samples [66, 71, 72]. All of the identified taxa are rare sequence types, representing typically less than 0.1% of sequences in the house dust samples. This low abundance at least challenges the usefulness of these specific taxa in analyses against health outcomes in the search of causally involved agents. The potential value of these candidate taxa will likely be restricted to their use as moisture damage indicators, contributing to a list of “moisture damage signatures” that may be defined with support of other sequencing studies in the future. Such effort could be considered the next generation of moisture damage indicator microbes, expanding the list that has been proposed in 1994 by Samson and colleagues in the era of cultivation [6] towards the use of DNA-based measurement approaches.

The limited number of homes and samples in this original moisture damage intervention study restricts broad conclusions on the identified microbial peculiarities and signatures of moisture damage. Samples of settled dust and floor dust were used as sample materials to analyze changes in the indoor microbiota following moisture damage interventions. We acknowledge that house dust samples have limitations in how well they reflect airborne exposures and potentially also in how well they reflect moisture conditions and changes of such conditions in buildings. A clear strength is the intervention design that was utilized to highlight potential moisture damage indicators. It is well known from studies of the built environment that the indoor microbiota are affected by and shaped by a multitude of factors relating to the outdoor environment, building characteristics and use, and occupants and their behavior [9, 73]. Thus, monitoring the same homes or at least the same families occupying homes in pre versus post intervention comparisons is powerful. Moreover, the candidate taxa of our study were in part replicated in a large cohort of homes with varying degrees of moisture damage. However, both of these studies were geographically restricted to Finland, its climatic, building and other specificities. Future studies carried out in other countries and climates will need to determine whether the findings of this study have wider implications in terms of their applicability to moisture damage conditions more broadly.

## Conclusions

This is one of the first studies to use culture-independent, high throughput sequencing to investigate the fungal and bacterial microbiota of severely moisture-damaged homes in a pre- versus post-renovation follow-up. We find that the impact of the intervention on microbial exposures for the occupants overall was subtle, and we recognize that more work remains to be done in order to support a quantitative and qualitative measurement of moisture damage using DNA-based approaches. However, several findings of this study should support future inquiries on the association between indoor microbiota and moisture damage using sequencing methods. For one, findings showed that richness decreased significantly during renovations, indicating that the number of different microbial taxa may be an important component of indoor exposures to consider, also in the context of moisture damage. Two, for bacteria we observed a clear concentration of moisture damage renovation associated taxa within the order *Actinomycetales*, which may provide a more focused target for future damp-building-related studies. Finally, this study demonstrates the utility of comparing pre and post intervention homes as a strategy for identifying moisture damage-associated microbial exposures.

## Addtional files

Additional file 1: The Lukas cohort; Microbiota of different types of house dust in moisture damaged homes; References. (DOC 33 kb)
Additional file 2: Table S1 to S17. Detailed table legends are provided in the individual excel sheets. (XLSX 139 kb)
Additional file 3: Figure S1. Mean relative abundance of the top 15 bacterial (A) and fungal (B) genera in dust samples from 41 moisture damage residences (floor dust living room (FDLR), floor dust form other rooms with moisture damage (FDOR), airborne settled dust (SD)). (TIFF 100 kb)
Additional file 4: Figure S2. Categorization of moisture damaged homes based on low (blue), medium (yellow) and high (red) fungal viable growth on building materials and impact on fungal Bray-Curtis-based beta-diversity in house dust of these homes. (PDF 10 kb)
Additional file 5: Figure S3. Regression coefficient plots for the slope of the relationship between OTU relative abundance and increasing severity of moisture damage or visible mold. The shaded areas enclose the 95% confidence interval and where the lower limit is above the 0-line the quantile of the OTU relative abundance is significantly higher in homes with higher damage/mold severity classification. Quantiles from the 50th (median) to 95th are presented; the confidence intervals were calculated with 0.05 quantile intervals using 200 permutations. Panel A-C, moisture damage; panel D-F, visible mold. *Aeromicrobium* sp. OTU (A,D); *Phycicoccus* sp. OTU (B,E); *Humicola nigrescens* (C,F). (TIFF 125 kb)
