# Supplementary material for: Indoor microbiota in severely moisture damaged homes and the impact of interventions
Source: Microbiome. 2017 Oct 13;5:138. doi: 10.1186/s40168-017-0356-5 (PMC5640920; doi:10.1186/s40168-017-0356-5)
Supplement: Additional file 1: — The Lukas cohort; Microbiota of different types of house dust in moisture damaged homes; References. (DOC 33 kb) [file 40168_2017_356_MOESM1_ESM.doc]

**SUPPLEMENTAL TEXT**

**The LUKAS cohort**

The replication stage of the study included children from LUKAS study consisting of two Finnish birth cohorts, LUKAS1 and LUKAS2 [1]. In LUKAS1 (N=214) equal numbers of pregnant mothers living in farms with livestock and mother’s living in rural areas but not in farms were recruited in the major local hospitals in eastern and middle Finland between September 2002-May 2004. LUKAS2 (N=228) is a random cohort extension to LUKAS1, in which all pregnant women with estimated delivery at Kuopio University Hospital between May 2004 and May 2005, were invited to join the study without selection by occupation or area of living. Mothers living in apartments were excluded to maintain housing conditions comparable with LUKAS1. Written informed consent was acquired from all LUKAS mothers. Ethical permission was granted by the Research Ethics Committee, Hospital District of Northern Savo.

***Building inspections, sampling and sample processing of house dust.*** All study homes were visited by a trained building engineer at mean age of 5 months of the study child and building assessments for moisture damage, dampness and mold were conducted using standardized protocols as described in detail in Karvonen et al. [1]. For the current analyses we used two three category variables reflecting observations of moisture damage and visible mold. ‘No damage’ was defined as no need for repair (class 0) or only cosmetic repair (classes 1). ‘Major damage’ was defined as either a need for repair of surface materials (class 2) with the area of damage ≥1 m2 or a need for repair of structural components (class 3) with the area of damage ≥0.1 m2, or a need for more extensive repair (classes 4 or 5). Other damage was classified as ‘minor damage’ [1]. During the home inspection, observed visible mold was also recorded in each room and categorized into three classes: no mold, spots of mold, and visible mold.

The dust samples were collected by the occupant from living room floor at of the participating study child. The sample was collected into a nylon sampling sock by vacuuming an area of 1 m2 from a rug for two minutes or in the absence of a rug, an area of 4 m^2^ from a smooth floor for two minutes. The dust samples were homogenized by sieving through sterile strainer, dried in desiccator and stored at -20°C until DNA extraction. Genomic DNA was extracted from 20mg of dust using bead beating method and chemagic DNA plant kit (Perkin Elmer) on the KingFisher DNA extraction robot.

***Sequencing and sequence processing of the LUKAS house dust microbiota.*** The bacterial/archaeal 16S rRNA gene V4 region was amplified using 515F/806R primers [2] and fungal ITS region by ITS1F/ITS2 primers [3]. These DNA amplicons were sequenced as 300 base pair paired-end reads with Illumina MiSeq V3 chemistry. The amplifications and sequencing were performed by LGC Genomics GmbH, Berlin, Germany. Sequence reads were merged with FLASH [4] and QIIME [5] was used for quality filtering, exclusion of chimeric sequences and further processing. Bacterial sequences were clustered into OTUs at 97% similarity using open-reference protocol (references= against the 16S rRNA gene database, greengenes, or ITS database Unite. OTUs representing less than 0.001% of the total sequences (minimum count of 83 and 93 sequences for bacteria and fungi, respectively) were excluded. Chloroplast (n=93) and mitochondrial (n=23) sequences were removed from the bacterial OTU table. For Fungi, the data processing was similar until the chimera removal step. We used FHiTINGS (Fungal High throughput Taxonomy Identification in NGS) to calculate taxa based OTU groups instead of clustering [6]. Taxonomic classification was obtained using the RDP classifier [7] for bacteria and FHiTINGS for fungi.

**Microbiota of different types of house dust in moisture damaged homes**

At the phylum level, *Proteobacteria* sequences were somewhat more abundant in airborne settled dust compared to floor dust, at the expense of *Firmicutes*. For fungi, Ascomycota – at the expense of Basidiomycota - were more abundant in settled dust (71%) compared to floor dust vacuumed from living (62%) and other moisture damaged rooms (49%) *(data not shown)*. We compared the mean relative abundances of the most prominent bacterial and fungal genera for the different sample types (Figure S1A and S1B). Not further defined genera within *Planococcaceae* family and *Lactococcus* genus were somewhat less abundant in settled dust compared to floor dust; in turn, several taxa were slightly more abundant in airborne settled dust, including *Micrococcus*, *Pseudomonas*, *Enterobacteriaceae* genera, and *Chryseobacterium*. For fungi, less *Candida*, *Cystofilobasidium* and *Russula*, and more *Cladosporium* and *Trichoderma* occurred in airborne settled dust compared to floor dust.

**REFERENCES**

1. Karvonen AM, Hyvärinen A, Roponen M, Hoffmann M, Korppi M, Remes S, von Mutius E, Nevalainen A, Pekkanen J. Confirmed moisture damage at home, respiratory symptoms and atopy in early life: a birth-cohort study. Pediatrics 2009;124(2):e329-38.

2. Caporaso JG, Lauber CL, Walters WA, Berg-Lyons D, Lozupone CA, Turnbaugh PJ, Fierer N, Knight R. Global patterns of 16S rRNA diversity at a depth of millions of sequences per sample. Proc. Natl. Acad. Sci. U. S. A. 2011;108(1):4516-4522.

3. Smith DP, Peay KG. Sequence depth, not PCR replication, improves ecological inference from next generation DNA sequencing. PLoS One 2014;9: e90234.

4. Magoc T, Salzberg SL. FLASH: fast length adjustment of short reads to improve genome assemblies. Bioinformatics 2011;27:2957-2963.

5. Caporaso JG, Kuczynski J, Stombaugh J, Bittinger K, Bushman FD, Costello EK, Fierer N, Pena AG, Goodrich JK, Gordon JI. QIIME allows analysis of high-throughput community sequencing data. Nature methods 2010;7:335-336.

6. Dannemiller KC, Reeves D, Bibby K, Yamamoto N, Peccia J. Fungal High‐throughput Taxonomic Identification tool for use with Next‐Generation Sequencing (FHiTINGS). J. Basic Microbiol. 2014;54:315-321.

7. Wang Q, Garrity GM, Tiedje JM, Cole JR. Naive Bayesian classifier for rapid assignment of rRNA sequences into the new bacterial taxonomy. Appl. Environ. Microbiol. 2007;73:5261-5267.
