# Supplementary figures and images for: Indoor microbiota in severely moisture damaged homes and the impact of interventions
Source: Microbiome. 2017 Oct 13;5:138. doi: 10.1186/s40168-017-0356-5 (PMC5640920; doi:10.1186/s40168-017-0356-5)

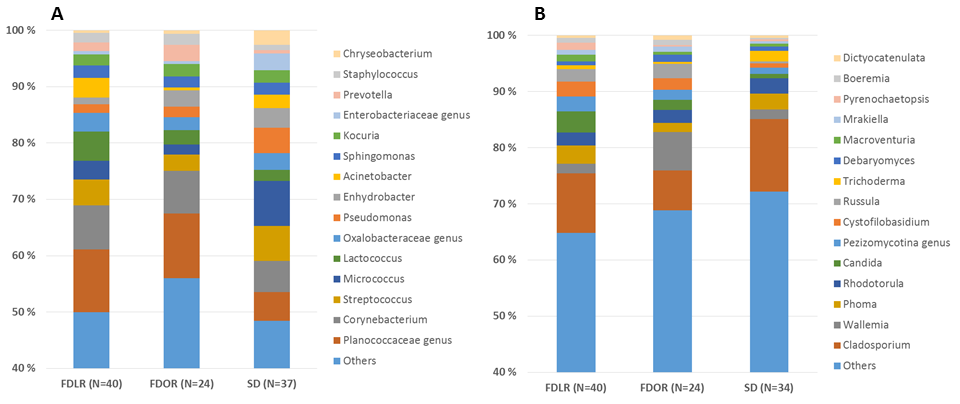

Supplement: Additional file 3: Figure S1. — Mean relative abundance of the top 15 bacterial (A) and fungal (B) genera in dust samples from 41 moisture damage residences (floor dust living room (FDLR), floor dust form other rooms with moisture damage (FDOR), airborne settled dust (SD)). (TIFF 100 kb) [file 40168_2017_356_MOESM3_ESM.tif]

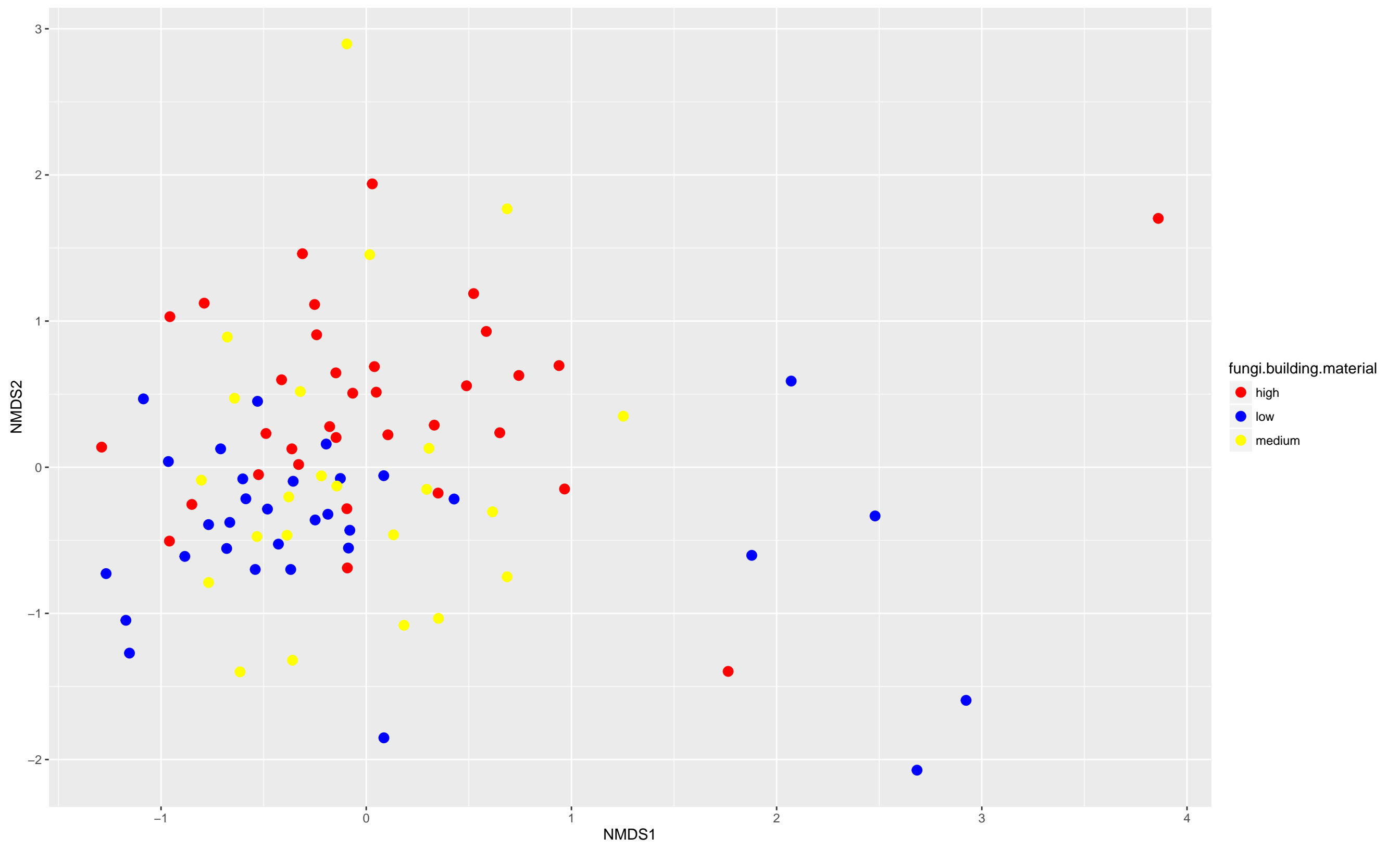

Supplement: Additional file 4: Figure S2. — Categorization of moisture damaged homes based on low (blue), medium (yellow) and high (red) fungal viable growth on building materials and impact on fungal Bray-Curtis-based beta-diversity in house dust of these homes. (PDF 10 kb) [file 40168_2017_356_MOESM4_ESM.pdf]

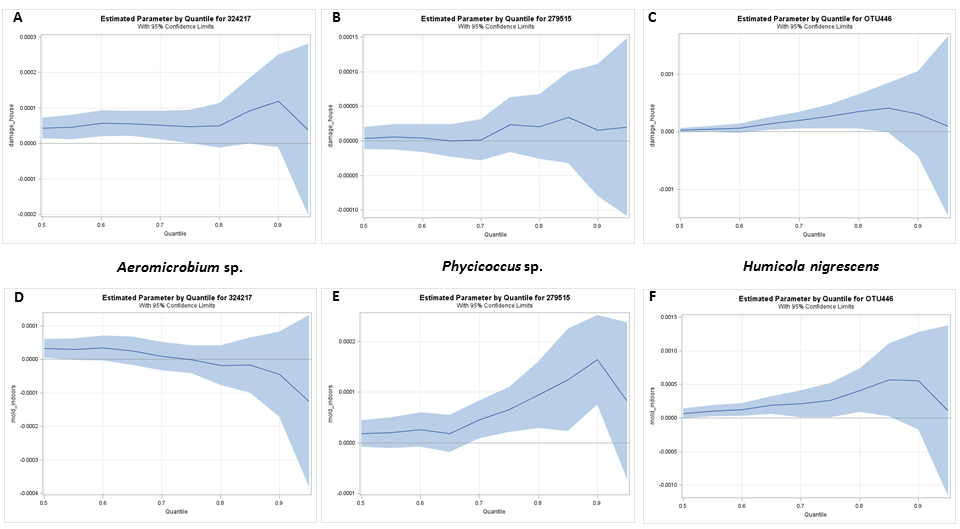

Supplement: Additional file 5: Figure S3. — Regression coefficient plots for the slope of the relationship between OTU relative abundance and increasing severity of moisture damage or visible mold. The shaded areas enclose the 95% confidence interval and where the lower limit is above the 0-line the quantile of the OTU relative abundance is significantly higher in homes with higher damage/mold severity classification. Quantiles from the 50th (median) to 95th are presented; the confidence intervals were calculated with 0.05 quantile intervals using 200 permutations. Panel A-C, moisture damage; panel D-F, visible mold. Aeromicrobium sp. OTU (A,D); Phycicoccus sp. OTU (B,E); Humicola nigrescens (C,F). (TIFF 125 kb) [file 40168_2017_356_MOESM5_ESM.tif]
